# Supplementary material for: Analysis of Crop Consumption Using Scatological Samples from the Red-Crowned Crane Grus japonensis in Eastern Hokkaido, Japan
Source: Animals (Basel). 2023 Oct 10;13(20):3167. doi: 10.3390/ani13203167 (PMC10603659; doi:10.3390/ani13203167)
Supplement: Supplementary file 1 [file animals-13-03167-s001.zip › animals-2553642-supplementary.pdf]

Supplementary files

# Analysis of crop consumption using scatological samples from red-crowned crane *Grus japonensis* in eastern Hokkaido, Japan

Ayaka Yokokawa[1], Kunikazu Momose[2], Dong Wenjing[1], Tatsuro Nakamura[1], Hiroko Iima[3], Kenichi Izumi[4], Yusuke Kawai[5], Tomoko Amano[6], Tatsuro Nakamura[1], Akira Sawada[6], Daiji Endoh[1], Nobuyoshi Nakajima[7] and Hiroki Teraoka[1,2]

[1]School of Veterinary Medicine, Rakuno Gakuen University, Ebetsu, Hokkaido, Japan; s21761102@stu.rakuno.ac.jp (A.Y.); dongwenjing_2015@163.com (W.D.); ky-upm@rakuno.ac.jp (N.T.); dendoh@rakuno.ac.jp (D.E.); hteraoka08@gmail.com (H.T.)
[2]NPO Red-crowned Crane Conservancy, Kushiro, Hokkaido, Japan; DZI00244@nifty.com (K.M.)
[3]Kushiro Zoo, 085–0201 Kushiro, Hokkaido, Japan; hiroko.iima@city.kushiro.lg.jp (H.I.); garrulaxcano-rus@yahoo.co.jp (T.Y.)
[4]College of Agriculture, Food and Environment Sciences, Rakuno Gakuen University, Ebetsu, Hokkaido 069-8501, Japan; izmken@rakuno.ac.jp (K.I.); amano@rakuno.ac.jp (T.A.)
[5]Laboratory of Toxicology, Department of Veterinary Medicine, Obihiro University of Agriculture and Veterinary Medi-cine, 2-11 Inada-cho Nishi, Obihiro 080-8555, Hokkaido, Japan; ykawai@obihiro.ac.jp (Y.K.)
[6]Biodiversity Division, National Institute for Environmental Studies, Tsukuba, Ibaraki 305-8506, Japan; naka-320@nies.go.jp (N.N.)
*Correspondence: hteraoka08@gmail.com

## **Table S1.** Individual information on intestinal contents of flying red-crowned cranes used in this study

| No. in Figure 1A | Stock No. | Stage | Sex | Collection date | Collection site | Reg. Promo. Bureau | No. in Figure 1A | Stock No. | Stage | Sex | Collection date | Collection site | Reg. Promo. Bureau |
|---|---|---|---|---|---|---|---|---|---|---|---|---|---|
| 1 | R134 | Adult | Male | 22-Mar-06 | Akan | Kushiro | 31 | R269 | Subadult | Female | 28-Mar-11 | Teshikaga | Kushiro |
| 2 | R136 | Subadult | Female | 2-May-06 | Urahoro | Tokachi | 32 | R272 | Adult | Female | 7-May-11 | Abashiri | Okhotsk |
| 3 | R143 | Adult | Male | 28-Jun-06 | Shibecha | Kushiro | 33 | R275 | Subadult | Male | 25-Jun-11 | Toyokoro | Tokachi |
| 4 | R146 | Subadult | Male | 19-Oct-06 | Bekkai | Nemuro | 34 | R281 | Subadult | Male | 2-Nov-11 | Taiki | Tokachi |
| 5 | R147 | Subadult | Female | 22-Oct-06 | Tsurui | Kushiro | 35 | R287 | Adult | Female | 1-Jan-12 | Shibecha | Kushiro |
| 6 | R148 | Subadult | Female | 23-Oct-06 | Nemuro | Nemuro | 36 | R317 | Adult | Female | 2-May-13 | Nemuro | Nemuro |
| 7 | R157 | Adult | Male | 19-Mar-07 | Shibecha | Kushiro | 37 | R474 | Adult | Male | 3-Sep-18 | Urahoro | Tokachi |
| 8 | R158 | Adult | Male | 30-Mar-07 | Akkeshi | Kushiro | 38 | R475 | Adult | Female | 3-Sep-18 | Urahoro | Tokachi |
| 9 | R160 | Adult | Male | 4-Apr-07 | Hamanaka | Kushiro | 39 | R476 | Juvenile | Female | 3-Sep-18 | Urahoro | Tokachi |
| 10 | R161 | Adult | Male | 9-May-07 | Nakashibetsu | Nemuro | 40 | R542 | Adult | Male | 30-Apr-20 | Shiranuka | Kushiro |
| 11 | R165 | Subadult | Male | 10-Jul-07 | Shibecha | Kushiro | 41 | R547 | Adult | Female | 29-Jul-20 | Akkeshi | Kushiro |
| 12 | R175 | Adult | Female | 16-Nov-07 | Urahoro | Tokachi | 42 | R548 | Juvenile | Female | 19-Aug-20 | Ikeda | Tokachi |
| 13 | R176 | Adult | Female | 20-Nov-07 | Tsurui | Kushiro | 43 | R549 | Adult | Male | 8-Sep-20 | Ikeda | Tokachi |
| 14 | R178 | Adult | Female | 27-Nov-07 | Kushiro City | Kushiro | 44 | R550 | Subadult | Female | 8-Sep-20 | Ikeda | Tokachi |
| 15 | R183 | Adult | Female | 11-Jan-08 | Shibecha | Kushiro | 45 | R551 | Adult | Male | 4-Oct-20 | Shibecha | Kushiro |
| 16 | R184 | Adult | Female | 14-Feb-08 | Hamanaka | Kushiro | 46 | R552 | Subadult | Female | 7-Oct-20 | Tsurui | Kushiro |
| 17 | R187 | Adult | Female | 11-Mar-08 | Hamanaka | Kushiro | 47 | R553 | Adult | Male | 9-Oct-20 | Tsurui | Kushiro |
| 18 | R196 | Subadult | Female | 21-Jul-08 | Bekkai | Nemuro | 48 | R555 | Adult | Male | 18-Oct-20 | Tsurui | Kushiro |
| 19 | R221 | Adult | Male | 27-Feb-09 | Shibecha | Kushiro | 49 | R556 | Subadult | Female | 26-Oct-20 | Tsurui | Kushiro |
| 20 | R223 | Adult | Female | 20-Mar-09 | Akkeshi | Kushiro | 50 | R558 | Juvenile | Male | 12-Nov-20 | Makubetsu | Tokachi |
| 21 | R225 | Adult | Male | 21-May-09 | Honbetsu | Tokachi | 51 | R560 | Adult | Female | 4-Dec-20 | Tsurui | Kushiro |
| 22 | R226 | Adult | Male | 2-Jun-09 | Bekkai | Nemuro | 52 | R561 | Subadult | Male | 5-Dec-20 | Ikeda | Tokachi |
| 23 | R241 | Subadult | Male | 22-Nov-09 | Teshikaga | Kushiro | 53 | R562 | Adult | Female | 8-Dec-20 | Nemuro | Nemuro |
| 24 | R243 | Adult | Female | 27-Jan-10 | Nemuro | Nemuro | 54 | R563 | Juvenile | Male | 17-Dec-20 | Tsurui | Kushiro |
| 25 | R245 | Adult | Male | 25-Feb-10 | Shibetsu | Nemuro | 55 | R564 | Juvenile | Male | 30-Dec-20 | Shiranuka | Kushiro |
| 26 | R246 | Adult | Male | 18-Mar-10 | Shibecha | Kushiro | 56 | R566 | Adult | Male | 10-Feb-21 | Onbetsu | Kushiro |
| 27 | R251 | Adult | Male | 24-May-10 | Shiranuka | Kushiro | 57 | R567 | Adult | Male | 2-Mar-21 | Shibecha | Kushiro |
| 28 | R262 | Adult | Female | 29-Nov-10 | Akkeshi | Kushiro | 58 | R569 | Adult | Female | 18-Mar-21 | Kushiro City | Kushiro |
| 29 | R264 | Subadult | Male | 16-Dec-10 | Teshikaga | Kushiro | 59 | R580 | Adult | Male | 5-Jun-21 | Ikeda | Tokachi |
| 30 | R265 | Subadult | Male | 20-Dec-10 | Kushiro City | Kushiro | 60 | - | Adult | - | 1-Aug-21 | Erimo | Hidaka |

Numbers for each crop indicate % of total reads from high-throughput sequencing. Reads less than 0.1% of the total read count were discarded. The numbers in the leftmost column number indicate the numbers in Figure 1A (Figure 1A No.). "Stock No." is used for each crane body in the freezer at Kushiro Zoo. "Reg. Promo. Bureau" (Regional Promotion Bureau) is one of the 14 promotion bureaus in Hokkaido Prefecture as an administrative unit covering some cities and towns.

**Table S2.** Individual information on red-crowned chick feces used in this study

| No. in Figure 1B | Stock No. | Stage | Sex | Collection date | Collection site | Reg. Promo. Bureau |
|---|---|---|---|---|---|---|
| 1 | 267 | Chick | Male | 25-Jun-16 | Ikeda | Tokachi |
| 2 | 268 | Chick | Female | 26-Jun-16 | Toyokoro | Tokachi |
| 3 | 270 | Chick | Female | 2-Jul-16 | Shiranuka | Kushiro |
| 4 | 271 | Chick | Male | 2-Jul-16 | Shiranuka | Kushiro |
| 5 | 272 | Chick | Male | 2-Jul-16 | Onbetsu | Tokachi |
| 6 | 280 | Chick | Female | 10-Jul-16 | Bekkai | Nemuro |
| 7 | 285 | Chick | Female | 16-Jul-16 | Hamanaka | Kushiro |
| 8 | 286 | Chick | Male | 16-Jul-16 | Hamanaka | Kushiro |
| 9 | 295 | Chick | Male | 24-Jun-17 | Ikeda | Tokachi |
| 10 | 296 | Chick | Female | 24-Jun-17 | Ikeda | Tokachi |
| 11 | 297 | Chick | Male | 24-Jun-17 | Ikeda | Tokachi |
| 12 | 299 | Chick | Female | 25-Jun-17 | Toyokoro | Tokachi |
| 13 | 305 | Chick | Female | 4-Jul-17 | Kushiro City | Kushiro |
| 14 | 306 | Chick | Male | 4-Jul-17 | Shiranuka | Kushiro |
| 15 | 307 | Chick | Female | 5-Jul-17 | Shibecha | Kushiro |
| 16 | 308 | Chick | Male | 5-Jul-17 | Shibecha | Kushiro |
| 17 | 309 | Chick | Male | 5-Jul-17 | Schibecha | Kushiro |
| 18 | 311 | Chick | Male | 7-Jul-17 | Bekkai | Nemuro |
| 19 | 316 | Chick | Male | 8-Jul-17 | Hamanaka | Kushiro |
| 20 | 317 | Chick | Female | 9-Jul-17 | Shintoku | Tokachi |
| 21 | 324 | Chick | Female | 13-Jul-18 | Kushiro City | Kushiro |
| 22 | 326 | Chick | Female | 7-Jul-18 | Shiranuka | Kushiro |
| 23 | 334 | Chick | Female | 14-Jul-18 | Teshikaga | Kushiro |
| 24 | 336 | Chick | Female | 14-Jul-18 | Teshikaga | Kushiro |
| 25 | 339 | Chick | Female | 15-Jul-18 | Shintoku | Tokachi |

Numbers for each crop indicate % of total reads from high-throughput sequencing. Reads less than 0.1% of the total read count were discarded. The numbers in the leftmost column number indicate the numbers in Figure 1B (Figure 1B No.). Banding No. is used for each crane body in the freezer at Kushiro Zoo. “Reg. Promo. Bureau” (Regional Promotion Bureau) is one of the 14 promotion bureaus in Hokkaido Prefecture as an administrative unit covering some cities and towns.

# **Table S3**. Specific primers for crops

| | Primer | Crop | Scientific name | Sequence | Purpose |
|---|---|---|---|---|---|
| 1 | Azuki F1 | Azuki bean | *Vigna angularis* | 5'-AGA ATC CTT TCA CCA AAA TTA CAG GAA-3' | First-PCR |
| 2 | Azuki R1 | | | 5'-ATC TAT TAC AAA ACT AGT CCC TTG TCA-3' | First-PCR |
| 3 | Azuki F2 | | | 5'-CAA TTC GAA GTT GAA GAA ACG ATG GA-3' | Nested-PCR |
| 4 | Azuki R2 | | | 5'-TAG GAA ATT GAT GAA AAT ACA AAT TCC-3' | Nested-PCR |
| 5 | Beet F2 | Sugar beet | *Beta vulgaris ssp. vulgaris* | 5'-TTA GAA TCG ATG AAC TCC GGA T-3′ | First/Nested-PCR |
| 6 | Beet R1 | | | 5'-TAG TGT GAT GTC CTT CCC CGT T-3' | First-PCR |
| 7 | Beet R2 | | | 5'-TCT CTA TCG GAA GTC TAT ATC TTC AT-3' | Nested-PCR |
| 8 | Cabbage F1 | Cabbage | *Brassica oleracea var. capitata* | 5'-GAA CTT CAA ATC TAC GAA GTA GA-3' | First-PCR |
| 9 | Cabbage R1 | | | 5'-AAA AGG GTA AGG TGT CAA ACA GAC-3' | First-PCR |
| 10 | Cabbage F2 | | | 5'-GAC TTA TAA TCT TTT TAC ATT GCA CAT-3' | Nested-PCR |
| 11 | Cabbage R2 | | | 5'-ACT CTG TTT TGT TCG CGT AAC CCA-3' | Nested-PCR |
| 12 | Carrot F1 | Carrot | *Daucus carota subsp. sativus* | 5'-AAA GCC CAT TCA ACT CCT TAT TAT CT-3' | First-PCR |
| 13 | Carrot R1 | | | 5'-TTA TGG ATC GGC TAA CAA CTA ATT TAA-3' | First-PCR |
| 14 | Carrot F2 | | | 5'-AGA GCA CAA TTT TTC TCT TAT GAC AA-3' | Nested-PCR |
| 15 | Carrot R02 | | | 5'-AAG ACC GAG GCG GAT AAA CG-3' | Nested-PCR |
| 16 | Corn F | Corn | *Zea mays* | 5'-GGA ACC TGC TAA GTG GTA ACT TC-3' | First-PCR |
| 17 | Corn R | | | 5'-GAC ACC CAC AGG TAG AAT GG-3' | First-PCR |
| 18 | Corn F02 | | | 5'-GTG GTT CTC AAA CTA GAA CCC AA-3' | Nested-PCR |
| 19 | Corn R2 | | | 5'-ATC CTC GTC CGA TTA ATC CAC TT-3' | Nested-PCR |
| 21 | Daizu F1 | Soybean | *Glycine max* | 5'-ACT CCC AAG TTT TCA GTC GGA AA-3' | First-PCR |
| 22 | Daizu R1 | | | 5'-TTA AAG ATA GAG AGG GAG GGT ATG-3' | First-PCR |
| 23 | Daizu F2 | | | 5'-GAA GTA GGG GCA TAG AAT CGA AA-3' | Nested-PCR qPCR |
| 24 | Daizu R2 | | | 5'-TCC TGG AAT TTT GAC GGA AGG ATT-3' | Nested-PCR qPCR |
| 25 | Hakusai F01 | Chinese cabbage | *Brassica rapa L. var. pekinensis* | 5'-ACG AAT CGA CCG TTC GAC TAT TTC T-3′ | First-PCR |
| 26 | Hakusai R01 | | | 5'-ACT GAG CTA TCC CGG CCA TTA CCG A-3' | First-PCR |
| 27 | Hakusai F02 | | | 5'-AGC AAA AAG GGG GAT ATG GCG GAA T-3' | Nested-PCR |
| 28 | Hakusai R02 | | | 5'-TTT AGG GAG TAG GGT TGG GGA TAG A-3' | Nested-PCR |
| 29 | Ingenmame F001 | Common bean | *Phaseolus vulgaris L.* | 5'-GTG GTA CTC CAG ATG CAC GCC TTG A-3' | First/Nested-PCR |
| 30 | Ingenmame R001 | | | 5'-TAT TGA GGG GGT GCC AGG GTG TGT T-3' | First-PCR |

| | Primer | Crop | Scientific name | Sequence | Purpose |
|---|---|---|---|---|---|
| 31 | Ingenmame R002 | | | 5'-TGG CCA AAG TCT CTG TGT GGA ACC A-3' | Nested-PCR |
| 32 | Jyagaimo F1 | Potato | *Solanum tuberosum L.* | 5'-TCT AAG CGT AAC TGG CTT TCT CTT-3' | First-PCR |
| 33 | Jyagaimo R1 | | | 5'-AAG TTG GGA AGT GAT CAT AGG CC-3' | First-PCR |
| 34 | Jyagaimo F2 | | | 5'-TCC CTA GTT GAA TGA TTC CCG ATC A-3' | Nested-PCR |
| 35 | Jyagaimo R2 | | | 5'-TTT CAT TCG CAT ACC GGT GGC A-3' | Nested-PCR |
| 36 | Kome F1 | Japanese rice | *Oryza sativa subsp. japonica* | 5'-CAT AAT AAA GAG AAC ATG CGA ATT TCT-3' | First-PCR |
| 37 | Kome R1 | | | 5'-TTA GAA GGG AGT TCC GCT AGC AA-3' | First-PCR |
| 38 | Kome F2 | | | 5'-CCG CTA GAC TAC ATA GAG TAA TGA A-3' | Nested-PCR |
| 39 | Kome R2 | | | 5'-TTT GGG TTC TAG TTC GAG AAC CG-3' | Nested-PCR |
| 40 | Komugi F1 | Wheat | *Triticum L.* | 5'-TTG AGC CTT GGT ATG GAA ACC T-3' | First-PCR |
| 41 | Komugi R1 | | | 5'-AGG GAC TTG AAC CCT CAC AAC T-3' | First-PCR |
| 42 | Komugi F2 | | | 5'-ACA AGG GGT TCT CGA ACT AGA A-3' | Nested-PCR |
| 43 | Komugi R2 | | | 5'-ACT CTC TCT TTG TCC TCG TCC-3' | Nested-PCR |
| 44 | Lettuce F01 | Lettuce | *Lactuca sativa* | 5'-GAA AAT GAG TAT GAG ACA TGA GGA-3′ | First-PCR |
| 45 | Lettuce R1 | | | 5'-TTA GGA ATT AAT CGG GAT TCT CAA A-3' | First-PCR |
| 46 | Lettuce F1 | | | 5'-TCT TTA GAA CCC CAT CTA TTT GAT T-3' | Nested-PCR |
| 47 | Lettuce R2 | | | 5'-AAC CAA ACG AAT TAT GCA GCA TCA ACT-3' | Nested-PCR |
| 48 | Oomugi F1 | Barley | *Hordeum vulgare L.* | 5'-TAA CTT CCA AAT TCA GAG AAA CCC-3' | First-PCR |
| 49 | Oomugi R1 | | | 5'-CTC ACA ACT TAA AAA GTC GTC G-3' | First-PCR |
| 50 | Oomugi F2 | | | 5'-GAA GGG ATT CTC GAA CTA GAA T-3' | Nested-PCR |
| 51 | Oomugi R2 | | | 5'-CAT GTA GAA TGG GAC TCT CTC-3' | Nested-PCR |
| 52 | Radish F1 | Japanese radish | *Raphanus sativus var. hortensis* | 5'-TTT GAA TTA GAC CGG AAA GCT TTT-3' | First-PCR |
| 53 | Radish R1 | | | 5'-TAA CTC AGG ATT TGG CTC AGG AT-3' | First-PCR |
| 54 | Radish F2 | | | 5'-TTT TGT TCT AAC CTC ATG CGA TTA T-3' | Nested-PCR |
| 55 | Radish R2 | | | 5'-ATG TTC TTC CTT TTC TCA TCG TTG TT-3' | Nested-PCR |
| 56 | Soba F1 | Buckwheat | *Fagopyrum esculentum* | 5'-AAA GGG CTA CCC TAA GAA AAG ACA AAA-3' | First-PCR |
| 57 | Soba R1 | | | 5'-AAT CTC AGT GTG ATA TAC TTT CTC CTT-3' | First-PCR |
| 58 | Soba F2 | | | 5'-ATG GAA TAG GCC TGT CGT ATA ATA TA-3' | Nested-PCR |
| 59 | Soba R2 | | | 5'-TTT CAT ACC GCA TAC CGT CGA ATT C-3' | Nested-PCR |
| 60 | Tomato F1 | Tomato | *Solanum lycopersicum* | 5'-ATG GGT CTC TCT TCC CTA GAA TGA A-3' | First-PCR |

| | Primer | Crop | Scientific name | Sequence | Purpose |
|---|---|---|---|---|---|
| 61 | Tomato R1 | | | 5'-ATT GGA ATT CTC ACA TTT AGT TGG AAG ATA-3' | First-PCR |
| 62 | Tomato F2 | | | 5'-ATA GCT TTA TCC AGT TTA GCG AGA T-3' | Nested-PCR |
| 63 | Tomato R2 | | | 5'-ATA GGA AGG AAT GGA GAA ATC GAG AAT A-3' | Nested-PCR |
| 64 | Alfalfa F1 | Alfalfa | *Medicago sativa* | 5'-GAG AAA CCC TGG AAT TAA AAA TGG GCA AT-3' | First-PCR |
| 65 | Alfalfa R1 | | | 5'-TCT TGA CAC ACC ACC CTA ATT TTA GGA A-3' | First/Nested-PCR |
| 66 | Alfalfa F2 | | | 5'-ATC CTG AGC CAA ATC CTT CTT TCC GAA AA-3' | Nested-PCR |

**Table S4.** Primers for high-throughput amplicon sequencing

| | Primer | Crop | Sequence |
|---|---|---|---|
| 1 | Beet Amplicon F | Sugar beet | 5′-TCG TCG GCA GCG TCA GAT GTG TAT AAG AGA CAG TTA GAA TCG ATG AAC TCC GGA T-3' |
| 2 | Beet Amplicon R | | 5'-GTC TCG TGG GCT CGG AGA TGT GTA TAA GAG ACA GTC TCT ATC GGA AGT CTA TAT CTT CAT-3' |
| 3 | Cabbage Amplicon F | Cabbage | 5'-TCG TCG GCA GCG TCA GAT GTG TAT AAG AGA CAG GAC TTA TAA TCT TTT TAC ATT GCA CAT-3′ |
| 4 | Cabbage Amplicon R | | 5'-GTC TCG TGG GCT CGG AGA TGT GTA TAA GAG ACA GAC TCT GTT TTG TTC GCG TAA CCC A-3' |
| 5 | Corn Amplicon F | Corn | 5'-TCG TCG GCA GCG TCA GAT GTG TAT AAG AGA CAG GTG GTT CTC AAA CTA GAA CCC AA-3' |
| 6 | Corn Amplicon R | | 5'-GTC TCG TGG GCT CGG AGA TGT GTA TAA GAG ACA GAT CCT CGT CCG ATT AAT CCA CTT-3' |
| 7 | Daizu Amplicon F | Soybean | 5'-TCG TCG GCA GCG TCA GAT GTG TAT AAG AGA CAG GAA GTA GGG GCA TAG AAT CGA AA-3' |
| 8 | Daizu Amplicon R | | 5'-GTC TCG TGG GCT CGG AGA TGT GTA TAA GAG ACA GTC CTG GAA TTT TGA CGG AAG GAT T-3' |
| 9 | Hakusai Amplicon F | Chinese cabbage | 5'-TCG TCG GCA GCG TCA GAT GTG TAT AAG AGA CAG AGC AAA AAG GGG GAT ATG GCG GAA T-3' |
| 10 | Hakusai Amplicon R | | 5'-GTC TCG TGG GCT CGG AGA TGT GTA TAA GAG ACA GTT TAG GGA GTA GGG TTG GGG ATA GA-3' |
| 11 | Ingenmame Amplicon F | Common bean | 5'-TCG TCG GCA GCG TCA GAT GTG TAT AAG AGA CAG GTG GTA CTC CAG ATG CAC GCC TTG A-3' |
| 12 | Ingenmame Amplicon R | | 5'-GTC TCG TGG GCT CGG AGA TGT GTA TAA GAG ACA GTG GCC AAA GTC TCT GTG TGG AAC CA-3' |
| 13 | Komugi Amplicon F | Wheat | 5'-TCG TCG GCA GCG TCA GAT GTG TAT AAG AGA CAG ACA AGG GGT TCT CGA ACT AGA A-3' |
| 14 | Komugi Amplicon R | | 5'-GTC TCG TGG GCT CGG AGA TGT GTA TAA GAG ACA GAC TCT CTC TTT GTC CTC GTC C-3' |
| 15 | Lettuce Amplicon F | Lettuce | 5'-TCG TCG GCA GCG TCA GAT GTG TAT AAG AGA CAG TCT TTA GAA CCC CAT CTA TTT GAT T-3' |
| 16 | Lettuce Amplicon R | | 5'-GTC TCG TGG GCT CGG AGA TGT GTA TAA GAG ACA GAA CCA AAC GAA TTA TGC AGC ATC AAC T-3' |
| 17 | Oomugi Amplicon F | Barley | 5'-TCG TCG GCA GCG TCA GAT GTG TAT AAG AGA CAG GAA GGG ATT CTC GAA CTA GAA T-3' |
| 18 | Oomugi Amplicon R | | 5'-GTC TCG TGG GCT CGG AGA TGT GTA TAA GAG ACA GCA TGT AGA ATG GGA CTC TCT C-3' |
| 19 | Radish Amplicon F | Japanese radish | 5'-TCG TCG GCA GCG TCA GAT GTG TAT AAG AGA CAG TTT TGT TCT AAC CTC ATG CGA TTA T-3' |
| 20 | Radish Amplicon R | | 5'-GTC TCG TGG GCT CGG AGA TGT GTA TAA GAG ACA GAT GTT CTT CCT TTT CTC ATC GTT GTT-3' |
| 21 | Tomato Amplicon F | Tomato | 5'-TCG TCG GCA GCG TCA GAT GTG TAT AAG AGA CAG ATA GCT TTA TCC AGT TTA GCG AGA T-3' |
| 22 | Tomato Amplicon R | | 5'-GTC TCG TGG GCT CGG AGA TGT GTA TAA GAG ACA GAT AGG AAG GAA TGG AGA AAT CGA GAA TA-3' |

# **Table S5.** Individual status of crop detection in intestinal contents of flying red-crowned cranes

| Figure 1A No. | Reference No. | Collection date | Collection site | Corn | Barely | Wheat | Soy bean | Radish | Cabbage /broccoli | Chinese cabbage | Lettuce /Prickly lettuce | Tomato |
|---|---|---|---|---|---|---|---|---|---|---|---|---|
| 3 | R143 | 28-Jun-06 | Shibecha | ○ | | | | | | | | |
| 4 | R146 | 19-Oct-06 | Bekkai | ○ | ○ | | | | | | | |
| 5 | R147 | 22-Oct-06 | Tsurui | ○ | | | | | | | | |
| 6 | R148 | 23-Oct-06 | Nemuro | ○ | | | | | | | | |
| 7 | R157 | 19-Mar-07 | Shibecha | ○ | | | | | | | | |
| 8 | R158 | 30-Mar-07 | Akkeshi | ○ | | | | | | | | |
| 9 | R160 | 4-Apr-07 | Hamanaka | ○ | | | | | | | ○ | |
| 10 | R161 | 9-May-07 | Nakashibetsu | ○ | | | | | | | | |
| 11 | R165 | 10-Jul-07 | Shibecha | ○ | | ○ | | | | | | |
| 12 | R175 | 16-Nov-07 | Urahoro | ○ | | | | | | | | |
| 13 | R176 | 20-Nov-07 | Tsurui | | | | ○ | | | | | |
| 14 | R178 | 27-Nov-07 | Kushiro | | | | | | | | ○ | |
| 15 | R183 | 11-Jan-08 | Shibecha | ○ | | | | | | | | |
| 16 | R184 | 14-Feb-08 | Hamanaka | | | | | | | ○ | | |
| 17 | R187 | 11-Mar-08 | Hamanaka | ○ | | | ○ | | | | | ○ |
| 18 | R196 | 21-Jul-08 | Bekkai | ○ | | | | | | | | |
| 20 | R223 | 20-Mar-09 | Akkeshi | ○ | | | | | | | | |
| 21 | R225 | 21-May-09 | Honbetsu | ○ | | | | | | | | |
| 22 | R226 | 2-Jun-09 | Bekkai | ○ | | | | | | | | |
| 23 | R241 | 22-Nov-09 | Teshikaga | ○ | | | | | | | | |
| 24 | R243 | 27-Jan-10 | Nemuro | ○ | | | | | | | | |
| 25 | R245 | 27-Feb-10 | Shibetsu | ○ | ○ | | | | | | | |
| 26 | R246 | 18-Mar-10 | Shibecha | | | | ○ | | | | | |
| 27 | R251 | 24-May-10 | Shiranuka | ○ | | | ○ | | | | | |
| 28 | R262 | 29-Nov-10 | Akkeshi | | | | | | | | | ○ |
| 29 | R264 | 16-Dec-10 | Teshikaga | ○ | | | | | | | | |
| 30 | R265 | 20-Dec-10 | Kushiro | ○ | | | | | | | | |
| 31 | R269 | 28-Mar-11 | Teshikaga | | | | | | | | ○ | |
| 32 | R272 | 7-May-11 | Abashiri | ○ | ○ | | | | ○ | | | |
| 33 | R275 | 25-Jun-11 | Toyokoro | ○ | | | | | | | | |
| 34 | R281 | 2-Nov-11 | Taiki | | | | | | | | ○ | ○ |
| 37 | R474 | 3-Sep-18 | Urahoro | ○ | | | | | | | | |
| 39 | R476 | 3-Sep-18 | Urahoro | | | ○ | | ○ | | ○ | ○ | |
| 40 | R542 | 30-Apr-20 | Shiranuka | ○ | | | | | | | | |
| 41 | R547 | 29-Jul-20 | Akkeshi | | | | | | | | ○ | |
| 45 | R551 | 4-Oct-20 | Shibecha | ○ | | ○ | | | | | | |
| 47 | R553 | 9-Oct-20 | Tsurui | ○ | | | | | | | ○ | |
| 48 | R555 | 18-Oct-20 | Tsurui | ○ | | | | | | | | ○ |
| 49 | R556 | 26-Oct-20 | Tsurui | ○ | | | | | | | ○ | |
| 51 | R560 | 4-Dec-20 | Tsurui | ○ | | | | | | | | |
| 52 | R561 | 5-Dec-20 | Ikeda | ○ | | ○ | | | | | | |
| 53 | R562 | 8-Dec-20 | Nemuro | ○ | | | | | | | | |
| 54 | R563 | 17-Dec-20 | Tsurui | | | ○ | | | | ○ | | |
| 55 | R564 | 30-Dec-20 | Shiranuka | ○ | | | | | | | | |
| 56 | R566 | 10-Feb-21 | Onbetsu | ○ | | | | | | | | |
| 58 | R569 | 18-Mar-21 | Kushiro | | | | ○ | | | | | |
| 59 | R580 | 5-Jun-21 | Ikeda | ○ | | | | | | | | |
| 60 | - | 1-Aug-21 | Erimo | ○ | | | | | | | | |

Because the read sequences of lettuce and prickly lettuce, a weed also found in eastern Hokkaido, completely matched, these were not considered a crop.

**Figure S1.** Numbers of crops detected in flying cranes (A) and chicks (B)

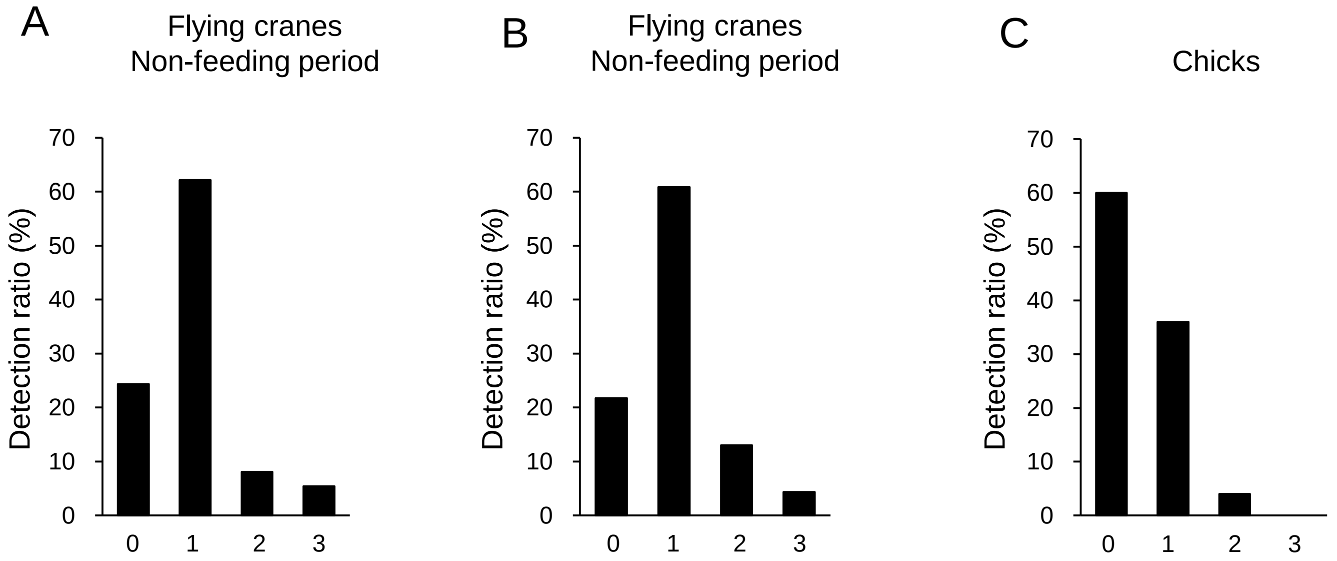


Numbers of crops detected in flying cranes and chicks. Percentages of the numbers of crops that were detected in each scatological sample of flying cranes (juveniles, subadults and adults) in non-feeding period (April – November, n = 37) (A) and feeding period (December – March, n = 23) (B) and chicks (n = 25) (C) are indicated. An adult crane found dead in Erimo (Number 60 in No. in Figure 1A) is included. Lettuce is not included because the read sequences of lettuce and prickly lettuce (*Lactuca scariola L.*) are the same.

**Figure S2.** Numbers of farms for three grains by city in eastern Hokkaido

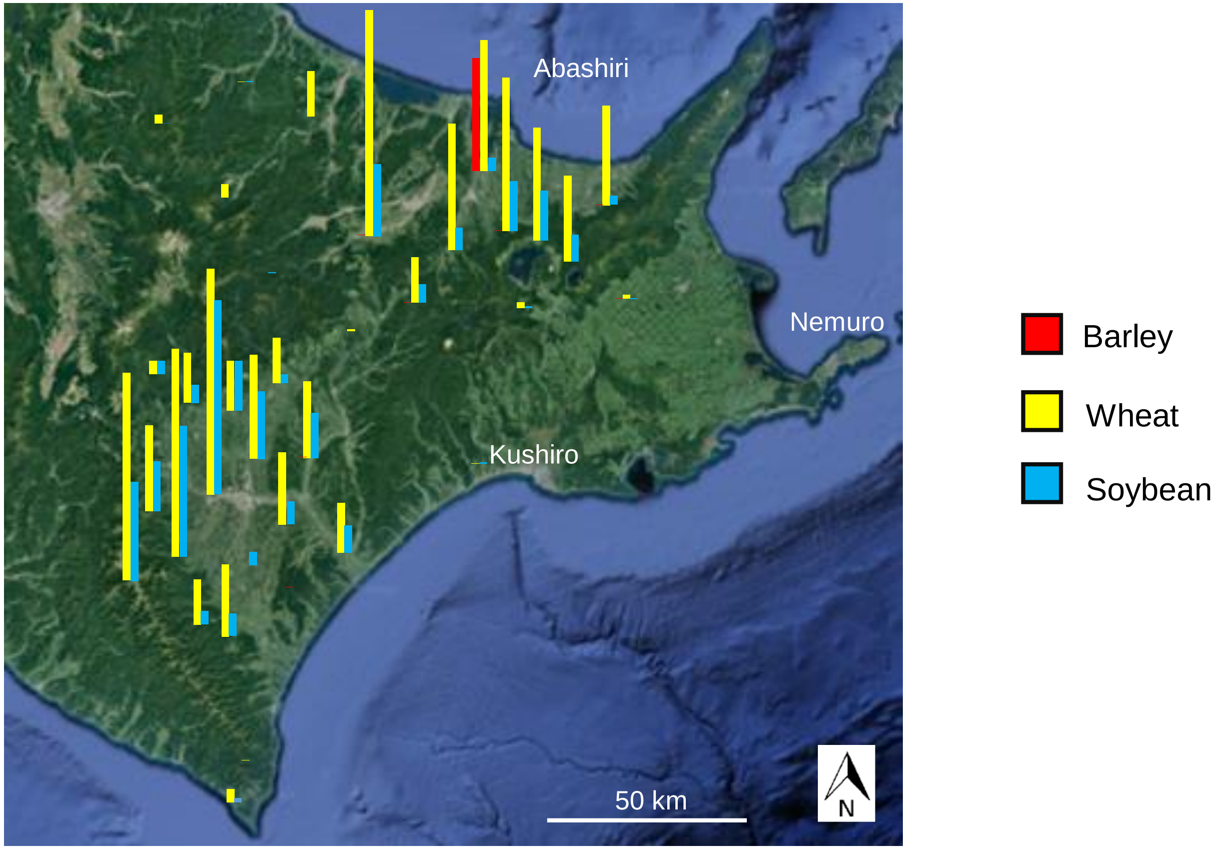


Numbers of farms for barley (red bar), wheat (yellow) and soybean (blue) are indicated by bar graphs. Data are based on 2020 Census of Agriculture and Forestry in Japan in 2020 (https://www.maff.go.jp/e/data/stat).

**Figure S3.** Numbers of farms for six vegetables by city in eastern Hokkaido

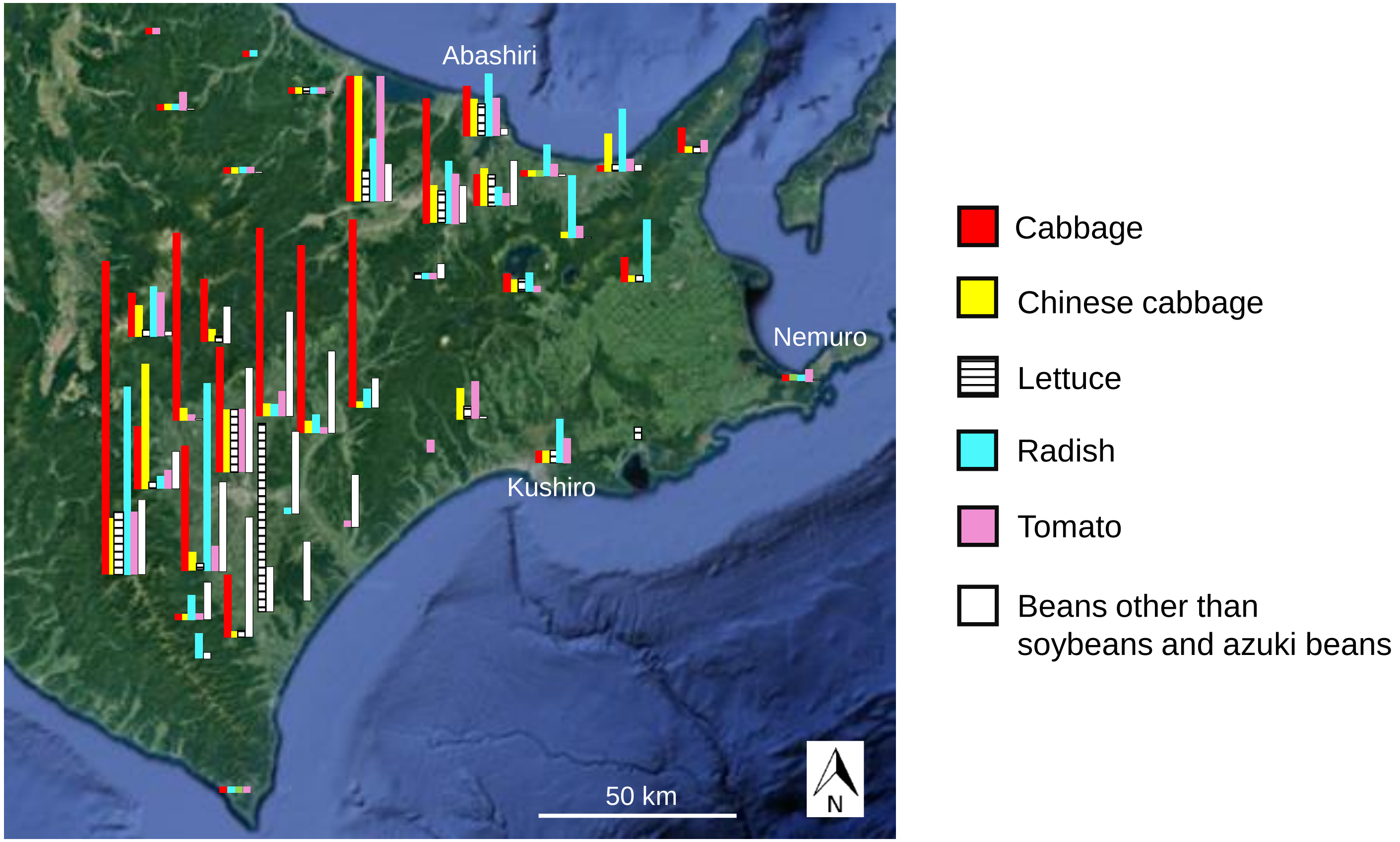


Numbers of farms for cabbage (red bar), Chinese cabbage (yellow), lettuce (lateral stripes), radish (light), tomato (purple) and beans other than soybeans and azuki beans (white) are indicated by bar graphs. Data are based on 2020 Census of Agriculture and Forestry in Japan in 2020 (https://www.maff.go.jp/e/data/stat/).

**Figure S4.** Monthly ingestion status of corn in flying red-crowned cranes

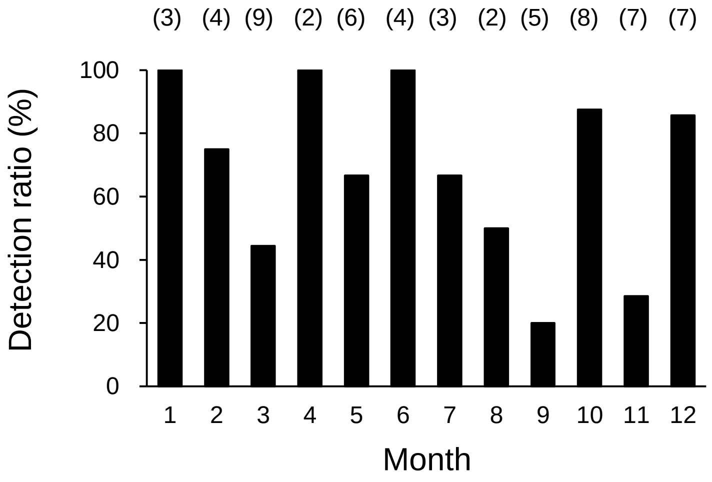


Monthly ingestion status of corn in flying cranes (juveniles, subadults and adults). Black bar indicates detection of corn for the month. Numbers in parentheses above bars indicate numbers of samples studied. Total of 60 cranes.

**Figure S5.** Corn kernels in compost pile of dairy cattle feces

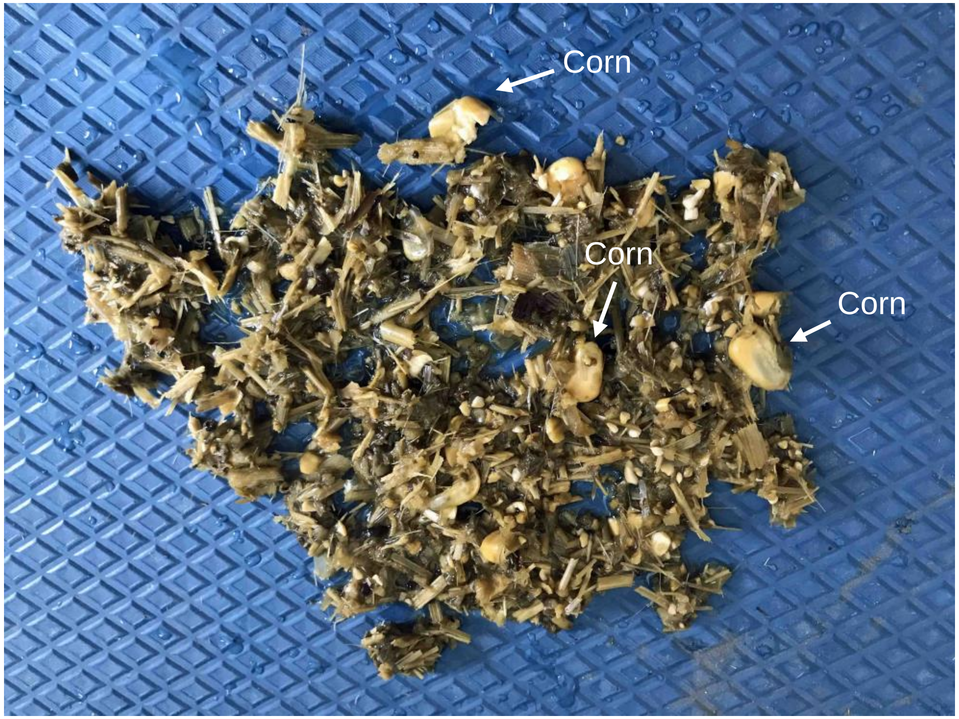


Three arrows indicate corn kernels.

**Table S6.** Detection of alfalfa by nested PCR and comparison with other crops

| Samples | City | Number in Fig. 1 | Stage | Date | Corn | Soybean | Lettuce | Alfalfa |
|---|---|---|---|---|---|---|---|---|
| R183 | Shibecha | 15 in A | Adults | 2008.1.18 | ○ | | | ○ |
| R564 | Shiranuka | 55 in A | Juveniles | 2020.12.30 | ○ | | | ○ |
| R566 | Onbetsu | 56 in A | Adults | 2021.2.10 | ○ | | | ○ |
| 305 | Kushiro | 13 in B | Chick | 2007.7.4 | ○ | ○ | | ○ |
| 324 | Kushiro | 21 in B | Chick | 2018.7.13 | | | ○ | ○ |
| 336 | Teshikaga | 22 in B | Chick | 2018.7.14 | ○ | | | ○ |

The collection sites of samples can be confirmed in Figure 1A and Table S1 (flying cranes) and Figure 1B and Table S2 (chicks).
